# A Delphi Study on the Healthcare Needs of Patients with Type 1 Diabetes during the Transition from Adolescence to Adulthood: Consensus among Patients, Primary Caregivers, and Healthcare Providers

**DOI:** 10.3390/ijerph18137149

**Published:** 2021-07-04

**Authors:** Yuehtao Chiang, Peikwei Tsay, Chiwen Chen, Chienlung Hsu, Hsingyi Yu, Chiwen Chang, Fusung Lo, Philip Moons

**Affiliations:** 1 School of Nursing, College of Medicine, Chang-Gung University, Taoyuan 33302, Taiwan; kanano@mail.cgu.edu.tw (H.Y.); cwc0166@gmail.com (C.C.); 2 Division of Pediatric Endocrinology & Genetics, Department of Pediatrics, Chang-Gung Memorial Hospital, Taoyuan 33305, Taiwan; 3 Department of Public Health and Center of Biostatistics, College of Medicine, Chang-Gung University, Taoyuan 33302, Taiwan; tsay@mail.cgu.edu.tw; 4 College of Nursing, National Yang Ming Chiao Tung University, Taipei 11221, Taiwan; chiwenchen@nycu.edu.tw; 5 Department of Information Management, Chang-Gung University, Taoyuan 33302, Taiwan; clhsu@mail.cgu.edu.tw; 6 Graduate Institute of Business and Management, Chang Gung University, Taoyuan 33302, Taiwan; 7 Healthy Aging Research Center, Chang Gung University, Taoyuan 33302, Taiwan; 8 Department of Visual Communication Design, Ming Chi University of Technology, New Taipei 24301, Taiwan; 9 Department of Nursing, Taoyuan Chang Gung Memorial Hospital, Taoyuan 33044, Taiwan; 10 Department of Nursing, Chang-Gung Memorial Hospital, Taoyuan 33305, Taiwan; 11 College of Medicine, Chung-Gung University, Taoyuan 33302, Taiwan; 12 Department of Public Health and Primary Care, KU Leuven-University of Leuven, 3000 Leuven, Belgium; philip.moons@kuleuven.be; 13 Institute of Health and Care Sciences, University of Gothenburg, 40530 Gothenburg, Sweden; 14 Department of Paediatrics and Child Health, University of Cape Town, Cape Town 7701, South Africa

**Keywords:** healthcare need, type 1 diabetes, Delphi methods

## Abstract

Patients with type 1 diabetes mellitus at the age of 16–25 face the challenges of the deterioration of disease control and accelerated exacerbation. Providing interventions that meet patient’s healthcare needs can reduce the impact and improve health outcome. The purpose of this study was to identify the healthcare needs of patients with type 1 diabetes during the adolescence to adulthood transition period from the perspectives of patients, parents and healthcare providers. A two-round Delphi study was conducted among 48 participants, and included 17 patients, 16 primary caregivers, and 15 healthcare providers. The central tendency and dispersion were computed to establish a consensus. Seventy-one healthcare needs were identified across five dimensions—technology, external support, internal support, management, and healthcare—and 56 were considered as important healthcare needs and with a moderate to high level of agreement. Meanwhile, patients, primary caregivers, and healthcare providers were found to display significantly different opinions (*p* < 0.05) for 23 healthcare needs. This study concluded the consensus of the healthcare needs of patients with type 1 diabetes mellitus during the adolescence to adulthood transition period from a systematic investigation. The findings can serve as reference for developing transitional intervention strategies.

## 1. Introduction

Type 1 diabetes is a chronic disease characterized by the autoimmune destruction of insulin-secreting B-cells and requires complex daily care regimens. It is the most common type of diabetes in youth under 18 years of age, although it may occur in all age groups [1,2]. Worldwide, the incidence and prevalence of type 1 diabetes have been increasing each year [2,3]. An analysis of the Taiwan National Health Insurance Research Database revealed that the incidence of type 1 diabetes among individuals aged 0–19 years increased from 4.84 per 100,000 population in 2005 to 5.17 per 100,000 population in 2014; the prevalence increased from 0.04% to 0.05% during the same period. Currently, in Taiwan, more than 11,000 patients have type 1 diabetes, accounting for approximately 0.6% of all diabetes cases [4]. The increased incidence and prevalence of type 1 diabetes among children and adolescents implies that the need for patient care during the transition from late adolescence to early adulthood will eventually increase.

Late adolescence to early adulthood represents an important stage of life—from being dependent to becoming independent. It is also a critical period for patients with chronic diseases as they transition from pediatric care to an adult self-care model [5,6]. This is a crucial time for glycemic control; optimal HbA1c control during this stage can significantly reduce macrovascular and microvascular complications [1]. However, the control of HbA1c is usually suboptimal or even poorer during this period [7,8]. Self-care for youth with type 1 diabetes during this transition period includes the management of increased insulin demand due to drastic hormonal changes and the unstable glucose levels potentially caused by alcohol and substance use [9,10]. Considering the transfer of care responsibilities, self-care is too complex and beyond the imagination of young patients. Thus, they feel conflicted, often swaying between independence and dependence [11,12]. With regard to interpersonal relationships, this period coincides with the transition of educational institutions—for example, from high school to university—leading to the dissolution and reestablishment of peer relationships. This poses pressure to the patients, leaving them feeling unsupported [11,13]. Additionally, the development of intimate and interpersonal relationships at the workplace are also novel aspects that young patients have rarely encountered in the past, and consequently, many patients feel frustrated [10,14]. In terms of the care system, most patients are required to transition from a pediatric to an adult care system, and both are completely different in terms of care culture and physician–patient interactions. For instance, appointments for adults in outpatient clinics often lack reminders against high-risk behaviors, such as the impact of sex, drinking, and excessive food intake on the disease. Therefore, patients tend to overlook the importance of self-care during this period [10,15]. Additionally, change in health insurance is a common concern of patients transitioning to adult healthcare. Patients in the transition period begin to worry about whether they can shoulder future financial burdens caused by the disease [11,12].

Youth with type 1 diabetes face many challenges and influences during the transition period. There is a great need for the support of the primary caregiver and healthcare provider. However, clinical experience has revealed that there are often many inconsistencies in their views on healthcare needs, and effective communication cannot be achieved. Only by understanding the different views can we further explore the causes of such differences, and develop strategies to ensure that patients, primary caregivers, and healthcare providers have consistent views on health needs, thus enhancing patients’ self-care motivation and improving the results of disease control [11,12,16]. Despite extensive research on care related to the transition period [11,12,13,17,18], few studies have investigated the extent of agreement regarding healthcare needs across patients, primary caregivers, and healthcare providers. The purpose of this study was to systemically identify the healthcare needs of patients with type 1 diabetes during the transition period from adolescence to adulthood by analyzing the perspectives of patients, primary caregivers, and healthcare providers. The results may be used as clinical care guidelines for adolescents and young adults with type 1 diabetes and serve as a reference for developing transitional intervention strategies.

## 2. Materials and Methods

### 2.1. Study Design

The Delphi method is a research technique in which a panel of experts participate in multiple discussions on a specific topic, anonymously in writing, to reach a consensus [19]. This method prevents situations such as conformity due to group pressure and authority, thus obtaining different levels of opinions [20]. Therefore, the Delphi method was used in this study. To maintain the rigor of the study, 12–15 experts were included in each group, and the targeted response rate was 70% or higher [21,22].

### 2.2. Participants and Setting

Based on previous qualitative research by the authors [11] and the relevant literature on care during the transition period [23,24], the selection criteria for stakeholders across the three categories were as follows: (1) patients: aged 16–25 years, diagnosed with type 1 diabetes for more than six months, who had no other metabolic disorders, chromosomal abnormalities, or catastrophic illness, and could speak Mandarin and Taiwanese; patients with autism or cognitive and language impairments were excluded. (2) Primary caregivers: individuals involved in the care of patients with type 1 diabetes aged 16–25 years for more than six months and could speak Mandarin and Taiwanese. (3) Healthcare providers: individuals with experience in practical clinical care for patients with type 1 diabetes aged 16–25 years who could provide relevant opinions. Purposive sampling was used to select qualified stakeholders from patients and primary caregivers in a medical center in northern Taiwan. The stakeholders from the healthcare provider group were selected from healthcare professionals in relevant fields across Taiwan and included physicians and nurses in pediatric and adult metabolism, diabetes educators, diabetes case managers, nutritionists, and nurse educators. Written consent forms were signed by all participants, and the legal representatives of patients who were minors.

A total of 48 participants were initially selected, including 17 patients, 16 primary caregivers, and 15 healthcare providers. After the first round, two patients and one primary caregiver informed the investigator that they would not have time to complete the second round of the questionnaire survey and withdrew consent. Therefore, their responses from the first-round questionnaires were not included in the statistical analysis.

### 2.3. Data Collection

Delphi surveys usually involve two to three rounds of questionnaire-based discussions [25]. In this study, two rounds of stakeholder consultations were conducted. The study was approved by the Chang Gung Medical Foundation Institutional Review Board (IRB No.: 201900242B0C601), and data were collected between April and July 2019. The researcher reached out to healthcare providers, inviting them to participate in this study. After they agreed, the researcher mailed the demographical information form and the first round of the Delphi questionnaire to the stakeholders. In addition, the patients and parents participants were recruited at the pediatric endocrinology and genetics clinic of a medical center in northern Taiwan. The response period for each round was two to three weeks. The data were compiled, analyzed, and modified within two weeks after collecting the responses for the first round. To achieve a consensus, the second round of the Delphi questionnaires were mailed to the participants.

### 2.4. Measures

Based on the results of a previous qualitative study conducted by the authors on the life experiences and healthcare needs of patients with type 1 diabetes aged 16–25 years [11] and a literature review, a questionnaire on healthcare needs during the transition period was drafted. The expert validity of the questionnaire was assessed. Five experts were invited to assess the scope, appropriateness, and validity of the questionnaire, and to modify the questionnaire. The final form comprised 71 healthcare needs categorized into five dimensions: 1. Technology (12 healthcare needs), which entailed the needs to establish or integrate interpersonal networks, disease knowledge, and care information into online platforms or mobile devices. 2. External support (19 healthcare needs), which measured the needs for external support in areas such as interpersonal relationships, employment, subsidies, policies, and associations. 3. Internal support (11 healthcare needs), which involved the needs for internal support at the psychological, emotional, stress relief, religious, and spiritual. 4. Management (19 healthcare needs), which referred to the needs to recognize type 1 diabetes and integrate a variety of care knowledge and methods to control the disease, prevent deterioration, and to maintain quality of life. 5. Healthcare (10 healthcare needs), which involved the content, space, quality, policy, and other healthcare service-related needs. A seven-point Likert scale was modified where 1 = not at all important, 2 = low important, 3 = slightly important, 4 = neutral, 5 = moderately important, 6 = very important and 7 = extremely important. A high score indicated that a need was crucial, while a low score indicated a need was of low importance [26]. The questionnaire is presented in Supplementary file.

### 2.5. Statistical Analysis

Descriptive statistics were computed using SPSS Version 21.0 (IBM Corp. Released 2012. IBM SPSS Statistics for Windows, Version 21.0. Armonk, NY, USA: IBM Corp.). Medians and interquartile ranges were calculated as the basis of central tendency and importance ranking, because the use of mean values to identify important items and rank the level of importance could be easily affected by extreme values, resulting in deviation from the center and causing errors. The lower and upper quartiles (Q1: 25% and Q3: 75%, respectively) were identified: Q1 > 5 and Q3 = 7 indicated that the healthcare needs were very important; Q1 = 5 and Q3 ≥ 6 indicated that the healthcare needs were important. To determine consensus, the quartile deviation (QD, QD = (Q3 − Q1)/2) of each item was obtained by analyzing the amount of discrete data. QD < 0.6 indicated that the stakeholder opinions on the item had a high level of agreement; a QD of 0.6–1.0 indicated a moderate level of agreement; and QD > 1.0 indicated a low level of agreement [27,28]. In this study, items considered important and with a moderate or higher level of agreement were categorized as “healthcare needs for which a consensus on importance was reached,” and the remaining items denoted “healthcare needs for which a consensus on importance was not reached.” In addition, the Kruskal–Wallis test was employed to determine whether significant differences existed between the stakeholder groups. The opinions of the three groups with significant differences (*p* < 0.05) were termed “healthcare needs for which there was a significant difference among stakeholders.” Post hoc analysis was subsequently performed, and Bonferroni’s correction was applied to analyze the differences between groups [27,28].

## 3. Results

### 3.1. General Characteristics of Participants

The response rate of the questionnaire was 100% in both rounds. The general characteristics of stakeholders who participated in the study are shown in Table 1. The percentages of males among the patients, primary caregivers and healthcare providers were 40.0%, 6.7% and 6.7%, respectively. Age ranges were 16–24 years (mean = 20.3 years) for patients, 36–60 years (mean = 49.1 years) for primary caregivers, and 35–53 years (mean = 43.3 years) for healthcare providers. The age of disease onset and duration of disease of the patients were in the ranges of 1–15 years (mean= 8 years) and 3–22 years (mean = 12.5 years), respectively. The duration of care for children with type 1 diabetes among the primary caregivers was within the range of 1.2–21 years (mean = 12.5 years), and the length of experience of healthcare providers in caring for patients with type 1 diabetes was within the range of 3–19 years (mean = 9.4 years). The proportions of patients with HbA1C < 8 mg/dL, 8 ≤ HbA1C < 10 mg/dL, 10 ≤ HbA1C < 12 mg/dL and HbA1C ≥ 12 mg/dL were 26.7%, 46.7%, 13.3% and 13.3%, respectively. The most common education level of the patients, primary caregivers and healthcare providers was university (73.3%), followed by senior high school (60.0%) and master’s degree (46.7%). All patients were single, whereas 14 (93.3%) primary caregivers and 12 (80%) healthcare providers were married. Furthermore, 73.3% of the patients and 46.7% of the healthcare providers were atheists, 60% of the primary caregivers were believers of Buddhism or Taoism. In terms of employment status, 53.3% of the patients were unemployed, 40.0% of the primary caregivers were homemakers, and the healthcare providers consisted of two physicians (13.3%), five nurses (33.3%), four health educators (26.7%) two case managers (13.3%), one nutritionist (6.7%) and one nurse educator (6.7%). For income, 40.0% of the patients and 33.3% of the primary caregivers had no income, and the majority (53.3%) of the healthcare providers had a monthly income of >TWD 50,000. Furthermore, 93.3% of the patients and healthcare providers, and all primary caregivers lived with their family.

### 3.2. Healthcare Needs of Patients with Type 1 Diabetes Aged 16–25 Years

#### 3.2.1. Healthcare Needs for Which Stakeholders Reached a Consensus on Importance

The second-round questionnaire results indicated that a consensus was reached on 56 healthcare needs across five dimensions, including 34 items that were considered by all three stakeholder groups as very important (Q1 > 5, Q3 = 7) and had a high level of agreement (QD < 0.6). The remaining 22 items were considered important by all three stakeholder groups (Q1 = 5, Q3 ≥ 6) with a moderate level of agreement (0.6 < QD ≤ 1). The results are presented in Table 2.

The 34 healthcare needs deemed very important included 3 in the technology dimension, 2 in external support, 5 in internal support, 17 in management, and 7 in healthcare. The technology dimension included three needs: (1) to develop a tailor-made app for type 1 diabetes; (2) develop an app that can analyze the relationships between blood glucose changes, diet, and insulin dosage, and provide recommendations; and (3) develop electronic guidelines on food calories and substitutions.

The external support dimension included two needs: (1) to provide organized and relevant information on type 1 diabetes; and (2) promote education on type 1 diabetes to reduce the stigmatization of patients with type 1 diabetes due to public misunderstanding.

The internal support dimension included five needs: (1) to assess and treat stress-induced sleep disorders; (2) being understood and accepted; (3) being recognized and encouraged for personal improvement; (4) to be given appropriate autonomy to learn independence and responsibility; and (5) the need for primary caregiver to replace control with supervision to reduce stress.

The management dimension included 17 needs: (1) to discuss the contents and goals of disease self-management; (2) discuss how to integrate disease care into daily life; (3) discuss diet management strategies that meet developmental needs; (4) discuss the accuracy of information on the internet; (5) provide step-by-step disease self-care instructions based on individual conditions; (6) plan and execute specific and feasible exercise programs based on patient preferences; (7) enhance disease-related knowledge based on individual needs; (8) differentiate between type 1 and type 2 diabetes and develop accurate understanding of own disease; (9) understand changes in disease progression and increase awareness regarding health maintenance; (10) understand the potential time, type, and severity of complications to increase crisis awareness and improve motivation for self-care; (11) understand the symptoms and care approaches for acute and chronic complications; (12) understand the purpose of treatment or medication adjustment to increase compliance; (13) understand the effects of hormonal changes during puberty on glycemic control to reduce frustration; (14) understand the possible impact of pregnancy on glycemic control and clarify misconceptions; (15) understand contraceptive measures to reduce the impact of unintended conception in young girls in the context of maternal and child health; (16) establish accurate knowledge of disease inheritance and clarify misconceptions to avoid unnecessary stress and fear; and (17) establish links to patient medical records that can be sent to other healthcare providers for reference when necessary or in the case of an emergency.

The healthcare dimension included seven needs: (1) the need for healthcare providers to replace accusations with gentle reminders and to avoid words that convey indifference and impatience; (2) to understand the respective concerns and needs of the primary caregiver and the patient during physician consultation; (3) provide practice opportunities when delivering healthcare instructions; (4) provide private consultation space to discuss private issues; (5) provide healthcare guidance that meets the cognitive development and disease needs of patients of all ages; (6) discuss topics on the transition from pediatric to adult care; and (7) establish a multidisciplinary diagnosis and management plan and system to reduce the back and forth across departments.

#### 3.2.2. Healthcare Needs for Which Stakeholders Did Not Reach a Consensus on Importance

The healthcare needs for which a consensus on importance was not reached included 15 items on three dimensions: technology, external support, and internal support. The results are presented in Table 3.

#### 3.2.3. Healthcare Needs for Which There Was a Significant Difference among Stakeholders

The opinions of the three stakeholder groups were significantly different regarding these healthcare needs, which included 23 items across the five dimensions (Table 2 and Table 3).

The results showed that there were significant differences between the opinions of patients and primary caregivers regarding 10 healthcare needs, including technology (one), external support (four), internal support (four), and management (one) dimensions. There was a significant difference in one of the needs in the technology dimension, to create a parent-only online chat room (*p* = 0.009). There were significant differences in four needs in the external support dimension: (1) to mediate parent–child conflicts and enhance mutual understanding (*p* = 0.05); (2) provide patients with skills to communicate with parents (*p* = 0.017); (3) provide employment counseling and consultation (*p* = 0.015); and (4) create type 1 diabetes card for patients (*p* = 0.001). There were significant differences in four needs in the internal support dimension: (1) to be given appropriate autonomy to learn independence and responsibility (*p* = 0.027); (2) provide resources for psychological counseling and consultation (*p* = 0.049); (3) provide stress management strategies (*p* = 0.017); and (4) assess emotional distress and provide coping skills (*p* = 0.012). There was a significant difference in one need in the management dimension, to discuss strategies to resist food cravings to improve the effectiveness of self-control with food (*p* = 0.006). All 10 needs were considered more important by the primary caregivers than by the patients.

There were significant differences between patients and healthcare providers in their opinions regarding three healthcare needs: one each in the internal support, management, and healthcare dimensions. There was a significant difference in the internal support dimension for the need to provide religious counseling resources (*p* = 0.040); only this item was considered less important by the healthcare providers than by the patients. In the management dimension, there was a significant difference in the need to establish accurate knowledge of disease inheritance and clarify misconceptions to avoid unnecessary stress and fear (*p* = 0.012). In the healthcare dimension, there was a significant difference in the need to develop virtual healthcare to reduce the impact of medical treatment on work or study (*p* = 0.001).

Furthermore, there were significant differences between primary caregivers and healthcare providers in their opinions regarding 16 healthcare needs across the five dimensions: technology (one), external support (seven), internal support (one), management (five), and healthcare (two). In the technology dimension, there was a significant difference in the need to create a chat room in which the patients can choose whether to share the contents with parents (*p* = 0.013). There were significant differences in seven needs in the external support dimension: (1) to provide employment counseling and consultation (*p* = 0.015); (2) cover insulin pump supplies in the health insurance plan (*p* = 0.001); (3) relax the disability handbook application criteria (*p* = 0.002); (4) include patients with type 1 diabetes under individuals with physical illness and provide learning assistance and resources *(p* = 0.015); (5) provide appropriate subsidies (*p* = 0.003); (6) create a type 1 diabetes card for patients (*p* = 0.001); and (7) change the name of the catastrophic illness card to something more positive to reduce labeling/stigmatization (*p* = 0.024). In the internal support dimension, there was a significant difference in the need to provide stress management strategies (*p* = 0.017). In the management dimension, there were significant differences in five needs: (1) to discuss the contents and goals of disease self-management (*p* = 0.010); (2) understand changes in disease progression and increase awareness regarding health maintenance (*p* = 0.022); (3) understand contraceptive measures to reduce the impact of unintended conception in young girls in the context of maternal and child health (*p* = 0.029); (4) establish accurate knowledge of disease inheritance and clarify misconceptions to avoid unnecessary stress and fear (*p* = 0.012); and (5) discuss strategies to resist food cravings to improve the effectiveness of self-control with food (*p* = 0.006). There were significant differences in two needs in the healthcare dimension: (1) to provide free or subsidized regular full body examinations (*p* = 0.006); and (2) develop virtual healthcare to reduce the impact of medical treatment on work or study (*p* = 0.001). All 16 needs were considered more important by the primary caregivers than by the healthcare providers.

## 4. Discussion

### 4.1. Consensus of Healthcare Needs

In the technology dimension, the items “develop a tailor-made app for type 1 diabetes,” “develop an app that can analyze the relationships between blood glucose changes, diet, and insulin dosage and provide recommendations,” and “develop electronic guidelines on food calories and substitutions” were very important healthcare needs. Meta-analyses showed that HbA1C dimensions were significantly lower in patients who used apps to manage blood glucose compared to that of the control group [29]. The use of mobile apps encouraged patients to engage in their own healthcare and increased their empowerment. However, the credibility, appropriateness, personalization, and accessibility of the app content were relatively insufficient. Payments were required for full access to all app functions, limiting the benefits of its use [30]. Currently, Chinese apps for both Android and iOS have several limitations. These include: a lack of type 1 diabetes-specific apps; a lack of personalization and inclusion of only general information; the recording function is limited to blood glucose, blood pressure, and body weight; and a lack of checklists regarding exercise, diet, and the management of emergencies that permit real-time recording. Therefore, the formulation of future intervention strategies should consider developing a customized app for type 1 diabetes that takes into account the needs of transitional patients.

In the external support dimension, during the transition from late adolescence to early adulthood, youth are particularly concerned about others’ opinions and criticism regarding themselves [6]. Stigmatization during interpersonal interactions is an impediment encountered by transition-period patients, who often do not know how to explain or clarify misconceptions in such scenarios [11]. This study showed that promoting “education on type 1 diabetes to reduce stigmatization of patients with type 1 diabetes due to public misunderstanding” was a very important healthcare need. Interpersonal difficulties caused by the illness can worsen the outcome of disease control and increase the psychological impact on the patients, which subsequently affects their perception of their own disease [11,31]. Therefore, establishing accurate information on type 1 diabetes among the general public, as well as fostering patients’ ability to clarify misconceptions so that they can gain more interpersonal support, should effectively reduce the psychological stress of patients and increase the effectiveness of disease control [32].

In the internal support dimension, “assess and treat stress-induced sleep disorders,” “being understood and accepted,” and “being recognized and encouraged for personal improvement” were very important healthcare needs. In addition to the pressures normally encountered during the transition from adolescence to early adulthood, patients with type 1 diabetes are also burdened with self-care related to the disease and often exhibit stress responses. Poor glycemic control is one of the biggest concerns during this period. Patients may even experience anticipatory anxiety that affects their sleep quality [11,12]. Therefore, it is necessary to recognize the source of psychological stress and provide personalized support, assessment, and treatment. Providing the youth with “appropriate autonomy to learn independence and responsibility” and the “need for primary caregiver to replace control with supervision to reduce stress” are also regarded as very important healthcare needs. Transition-period patients are usually willing to learn to become responsible for their own disease. However, disease care is complex. While they want their parents to be involved, the patients are unsure about expressing their needs because they fear being controlled by their parents [11,18]. Therefore, improvement in parent involvement and the provision of communication and consultation opportunities for parents and young patients are also topics that merit attention in clinical care.

In the management dimension, healthcare needs at this dimension had the highest level of agreement. Patients, primary caregivers, and healthcare providers all agreed to assume responsibility for disease management during this period. The establishment of accurate disease knowledge and the cultivation of disease management capabilities were very important healthcare needs, which included the following: “discuss the contents and goals of disease self-management,” “discuss diet management strategies that meet developmental needs,” and “enhance disease-specific knowledge, such as the progression and impact of comorbidities, the impact of pregnancy, inheritance, and hormones on blood glucose, and the approach and purpose of medication adjustment.” These could help patients avoid unnecessary stress and fear, improve their motivation for self-care, and increase compliance, thereby improving management effectiveness and health outcomes. These findings corroborate the results reported by Ersig et al. [12], Garvey et al. [10], and Sheehan et al. [33]. Moreover, due to the proliferation of information on the Internet, patients need to be assisted with assessing the sources of disease-related information. In this context, the healthcare providers’ need to “discuss the accuracy of information on the Internet” had a high level of agreement among stakeholders. Similar conclusions were drawn in the study by Vo et al. [30]. Compared with the childhood period, transition-period patients encounter an increased amount of learning and social interactions, leading to a faster pace of life. Disease care is often sacrificed to maintain balance due to competitive needs, but this also increases the risk of acute and chronic complications [11,31]. Therefore, it is a very important healthcare need and a goal to “discuss how to integrate disease care into daily life.”

In the medical dimension, it has been found that healthcare providers often adopt the adult care model for transition-period patients when delivering healthcare instructions in clinical settings. For instance, they explain or provide health education leaflets or resources, but seldom demonstrate the actual operation process or assess the intervention results. This study showed that providing “practice opportunities when delivering healthcare instructions” constituted a very important healthcare need and could be used as a reference when providing healthcare instructions in the future. Stakeholders also agreed on the importance of discussing “topics on the transition from pediatric to adult care” and developing “virtual healthcare to reduce the impact of medical treatment on work or study.” Therefore, these two healthcare needs should not be neglected.

### 4.2. Discrepancies between Patients, Parents, and Healthcare Providers

The healthcare needs of this category spanned across all five dimensions. A higher agreement level was reached between patients and healthcare providers than between patients and primary caregivers. Notably, primary caregivers placed more importance on healthcare needs compared with patients and healthcare providers. There remained many uncertainties among parents regarding the transition process of adolescents. The studies by Ersig et al. [12] and Gabele et al. [34] showed that parents were uncertain about (1) whether adolescent self-management led to immediate and long-term diabetes complications, such as hypoglycemia, amputation, or blindness; (2) whether adolescents could take responsibility and adjust their lives and monitor their blood glucose; and (3) whether adolescents could pay for their own medical expenses and continue with follow ups after the transition period. As primary caregivers also play a crucial role in the transition period, future research can explore the transitional care experiences of primary caregivers and provide them with appropriate interventions. This should ensure comprehensive care for patients with type 1 diabetes and improve the effectiveness of care during the transition period.

There are several limitations in this study. First, the post hoc analysis was used to find out the differences in healthcare needs opinions among groups. After post hoc analysis, there may be cases where the sample does not conform to the distribution of the population or the assumption of normal distribution. Although the Bonferroni correction, as a control method of the conservative family-wise error rate, can reduce the false inference of a type I error statistical test, it will increase the probability of a type II error. Second, the duration of disease of the patients ranged from 3 to 22 years; therefore, patients with different duration of type 1 diabetes may exhibit different healthcare needs. Finally, the present findings may be representative of the Taiwanese situation, therefore expanding the current knowledge from Western societies. Thus, the consensus-based results proposed by this study should be interpreted with caution and subjected to further evaluation of clinical practicability before application to countries with different social contexts.

## 5. Conclusions

These findings will enable healthcare providers to understand the healthcare needs of patients with type 1 diabetes aged 16–25 years during the transition period. The results can provide empirical guidance for clinical care and serve as a reference for developing intervention strategies. Regarding healthcare needs for which a consensus on their importance was reached, healthcare providers should determine whether these needs have been met and develop relevant intervention strategies to fill any gaps. In the case of healthcare needs exhibiting a major disagreement among stakeholders—particularly for dimensions with significant discrepancies from primary caregivers versus those from patients and healthcare providers—the reasons for disagreement and expectations should be understood to improve the effectiveness of communication between patients, primary caregivers, and healthcare providers.

## Figures and Tables

**Table 1:** Characteristics of the study participants.

	Group A Patients (*n* = 15)	Group B Primary Caregivers (*n* = 15)	Group C Healthcare Providers (*n* = 15)
**Gender, *n* (%)**			
Male	6 (40.0%)	1 (6.7%)	1 (6.7%)
Female	9 (60.0%)	14 (93.3%)	14 (93.3%)
Age (yrs.), mean (range)	20.3 (16.0–24.0)	49.1 (36.0–60.0)	43.3 (35.0–53.0)
**Age of disease onset (yrs.), mean (range), quartiles**	8.0 (1.0–15.0)	-	-
	Q1 = 5.0		
	Q2 = 7.0		
	Q3 = 13.0		
**Duration of disease/care (yrs.) mean (range), quartiles**	12.5 (3.0–22.0)	12.5 (1.2–21.0)	9.4 (3.0–19.0)
	Q1 = 9.0	Q1 = 9.0	Q1 = 5.3
	Q2 = 10.0	Q2 = 11.0	Q2 = 9.0
	Q3 = 19.0	Q3 = 19.0	Q3 = 14.0
**HbA1ca % (mmol/mol), *n* (%)**			
<8(64)	4 (26.7)	-	-
=8(64)~ < 10(86)	7 (46.7)	-	-
=10(86)~12(108)	2 (13.3)	-	-
≥12(108)	2 (13.3)	-	-
**Education level, *n* (%)**			
Junior high school	1 (6.7)		
Senior high school	3 (20.0)	9 (60.0)	1 (6.7)
University	11 (73.3)	3 (20.0)	6 (40.0)
Master’s degree	0 (0.0)	1 (6.7)	7 (46.7)
Doctoral degree	0 (0.0)	0 (0.0)	1 (6.7)
**Marital status, *n* (%)**			
Single	15 (100.0)	0 (0.0)	3 (20.0)
Married	0 (0.0)	14 (93.3)	12 (80.0)
Divorced	0 (0.0)	1 (6.7)	0 (0.0)
**Religious belief, *n* (%)**			
Atheism	11 (73.3)	5 (33.3)	7 (46.7)
Buddhism or Taoism	3(20.0)	9 (60.0)	6 (40.0)
Christianity or Catholicism	1 (6.7.0)	1 (6.7)	1 (6.7)
Others	0	0	1 (6.7)
**Employment status, *n* (%)**			
Unemployed	8 (53.3)	2 (13.3)	0 (0.0)
Farmer	1 (6.7)	0 (0.0)	0 (0.0)
Artisan	0 (0.0)	2 (13.3)	0 (0.0)
Merchant	1 (6.7)	2 (13.3)	0 (0.0)
Healthcare	0 (0.0)	1 (6.7)	15 (100.0)
Physician	-	-	2 (13.3)
Nurse			5 (33.3)
Health educator			4 (26.7)
Case manager			2 (13.3)
Nutritionist			1 (6.7)
Nurse educator			1 (6.7)
Service industry	5 (33.3)	2 (13.3)	0 (0.0)
Homemaker	0 (0.0)	6 (40.0)	0 (0.0)
**Personal financial status, *n* (%)**			
No income	6 (40.0)	5 (33.3)	0 (0.0)
<TWD 10,000/month	4 (26.7)	0 (0.0)	0 (0.0)
TWD 20,000 to <30,000/month	2 (13.3)	3 (20)	0 (0.0)
TWD 30,000 to <50,000/month	2 (13.3)	5 (33.3)	7 (46.7)
>TWD 50,000/month	1 (6.7)	2 (13.3)	8 (53.3)
**Residential status, *n* (%)**			
Living with family	14 (93.3)	15 (100)	14 (93.3)
Renting	1 (6.7)	0 (0.0)	0 (0.0)
Owner	0 (0.0)	0 (0.0)	1 (6.7)

**Table 2:** Analysis of differences across stakeholder groups regarding healthcare needs for which a consensus on importance was reached.

Healthcare Need	TOTAL (*n* = 45)	GROUP A: Patients (*n* = 15)	GROUP B: Caregivers (*n* = 15)	GROUP C: Healthcare Providers (*n* = 15)	*p*-Value Post Hoc
	Median (Q1, Q3) QD	Median (Q1, Q3) QD	Median (Q1, Q3) QD	Median (Q1, Q3) QD	
**1. Technology dimension**					
Develop a tailor-made app for type 1 diabetes	7 (6,7) 0.5 a,b	7 (6,7) 0.5 a,b	7 (7,7) 0 a,b	7 (6,7) 0.5 a,b	0.205
Develop an app that can analyze the relationships between blood glucose changes, diet, and insulin dosage and provide recommendations	7 (7,7) 0 a,b	7 (7,7) 0 a,b	7 (7,7) 0 a,b	7 (7,7) 0 a,b	0.504
Develop electronic guidelines on food calories and substitutions	7 (7,7) 0 a,b	7 (6,7) 0.5 a,b	7 (7,7) 0 a,b	7 (7,7) 0 a,b	0.313
Develop electronic diet and exercise journals	7 (6,7) 0.5 a,b	7 (5,7) 1 c,d	7 (7,7) 0 a,b	7 (6,7) 0.5 a,b	0.262
Develop an electronic journal for instant recording of symptoms, scenarios, and management of acute complications	7 (6,7) 0.5 a,b	7 (5,7) 1 c,d	7 (6,7) 0.5 a,b	6 (6,7) 0.5 a,b	0.570
Create an anonymous chat room for physicians and patients	6 (5,7) 1 c,d	6 (5,7) 1 c,d	7 (6,7) 0.5 a,b	5 (5,6) 0.5 c,b	0.051
Create an online portal for posting questions about type 1 diabetes	7 (6,7) 0.5 a,b	7 (5,7) 1 c,d	7 (6,7) 0.5 a,b	6 (6,7) 0.5 a,b	0.164
Create a type 1 diabetes knowledge network	7 (7,7) 0 a,b	7 (5,7) 1 c,d	7 (7,7) 0 a,b	7 (6,7) 0.5 a,b	0.150
**2. External support dimension**					
Provide organized and relevant information on type 1 diabetes	7 (6,7) 0.5 a,b	7 (6,7) 0.5 a,b	7 (7,7) 0 a,b	7 (6,7) 0.5 a,b	0.373
Promote education on type 1 diabetes to reduce stigmatization of patients with type 1 diabetes due to public misunderstanding	7 (7,7) 0 a,b	7 (7,7) 0 a,b	7 (7,7) 0 a,b	7 (6,7) 0.5 a,b	0.056
Need for a middleman to remind parents to learn to let go	6 (6,7) 0.5 a,b	7 (5,7) 1 c,d	7 (6,7) 0.5 a,b	6 (5,7) 1 c,d	0.316
Hold seminars for parents to share the skills of letting go	6 (6,7) 0.5 a,b	6 (5,7) 1 c,d	6 (6,7) 0.5 a,b	7 (6,7) 0.5 a,b	0.767
Mediate parent–child conflicts and enhance mutual understanding	7 (6,7) 0.5 a,b	6 (5,7) 1 c,d	7 (7,7) 0 a,b	7 (6,7) 0.5 a,b	0.005 B > A
Provide patients with skills to communicate with parents	7 (6,7) 0.5 a,b	6 (5,7) 1 c,d	7 (7,7) 0 a,b	7 (6,7) 0.5 a,b	0.017 B > A
Provide employment counseling and consultation	7 (5,7) 1 c,d	6 (5,7) 1 c,d	7 (7,7) 0 a,b	6 (5,7) 1 c,d	0.015 B > A,C
Hold employment seminars to share precautions and adjustment experience during job hunting and employment	6 (6,7) 0.5 a,b	7 (5,7) 1 c,d	7 (6,7) 0.5 a,b	6 (5,7) 1 c,d	0.121
Diversify the activities organized by diabetes associations to meet the needs of patients from different age groups	6 (5,7) 1 c,d	5 (5,7) 1 c,d	7 (6,7) 0.5 a,b	7 (6,7) 0.5 a,b	0.087
Cover insulin pump supplies in the health insurance plan	7 (6,7) 0.5 a,b	7 (5,7) 1 c,d	7 (7,7) 0 a,b	6 (5,6) 0.5 b,c	0.001 B > C
Relax the disability handbook application criteria	7 (5,7) 1 c,d	7 (6,7) 0.5 a,b	7 (7,7) 0 a,b	5 (5,6) 0.5 b,c	0.002 B > C
Include patients with type 1 diabetes under individuals with physical illness and provide learning assistance and resources	6 (6,7) 0.5 a,b	7 (6,7) 0.5 a,b	7 (6,7) 0.5 a,b	6 (5,6) 0.5 b,c	0.015 B > C
**3. Internal support dimension**					
Assess and treat stress-induced sleep disorders	6 (6,7) 0.5 a,b	6 (6,7) 0.5 a,b	7 (6,7) 0.5 a,b	6 (6,7) 0.5 a,b	0.505
Being understood and accepted	7 (6,7) 0.5 a,b	7 (6,7) 0.5 a,b	7 (7,7) 0 a,b	7 (6,7) 0.5 a,b	0.341
Being recognized and encouraged for personal improvement	7 (6.5,7) 0.25 a,b	7 (7,7) 0 a,b	7 (7,7) 0 a,b	7 (6,7) 0.5 a,b	0.223
Given appropriate autonomy to learn independence and responsibility	7 (6,7) 0.5 a,b	7 (6,7) 0.5 a,b	7 (7,7) 0 a,b	7 (6,7) 0.5 a,b	0.027 B > A
Need for primary caregiver to replace control with supervision to reduce stress	7 (6,7) 0.5 a,b	7 (6,7) 0.5 a,b	7 (6,7) 0.5 a,b	6 (6,7) 0.5 a,b	0.393
Provide resources for psychological counseling and consultation	7 (6,7) 0.5 a,b	7 (5,7) 1 c,d	7 (7,7) 0 a,b	7 (6,7) 0.5 a,b	0.049 B > A
Provide stress management strategies	7 (6,7) 0.5 a,b	6 (5,7) 1 c,d	7 (7,7) 0 a,b	6 (6,7) 0.5 a,b	0.017 B > A,C
**4. Management dimension**					
Discuss the contents and goals of disease self-management	7 (6,7) 0.5 a,b	7 (6,7) 0.5 a,b	7 (7,7) 0 a,b	6 (6,7) 0.5 a,b	0.010 B > C
Discuss how to integrate disease care into daily life	7 (6,7) 0.5 a,b	7 (6,7) 0.5 a,b	7 (7,7) 0 a,b	7 (6,7) 0.5 a,b	0.053
Discuss diet management strategies that meet developmental needs	7 (6,7) 0.5 a,b	7 (6,7) 0.5 a,b	7 (7,7) 0 a,b	7 (6,7) 0.5 a,b	0.308
Discuss the accuracy of information on the internet	7 (6,7) 0.5 a,b	7 (6,7) 0.5 a,b	7 (6,7) 0.5 a,b	7 (6,7) 0.5 a,b	0.889
Provide step-by-step disease self-care instructions based on individual conditions	7 (6,7) 0.5 a,b	7 (6,7) 0.5 a,b	7 (7,7) 0 a,b	6 (6,7) 0.5 a,b	0.207
Plan and execute specific and feasible exercise programs based on patient preferences	7 (6.5,7) 0.25 a,b	7 (7,7) 0 a,b	7 (7,7) 0 a,b	7 (6,7) 0.5 a,b	0.110
Enhance disease-related knowledge based on individual needs	7 (6,7) 0.5 a,b	7 (7,7) 0 a,b	7 (6,7) 0.5 a,b	7 (6,7) 0.5 a,b	0.268
Differentiate between type 1 and type 2 diabetes and develop accurate understanding of own disease	7 (6,7) 0.5 a,b	7 (7,7) 0 a,b	7 (7,7) 0 a,b	7 (6,7) 0.5 a,b	0.111
Understand changes in disease progression and increase awareness regarding health maintenance	7 (6,7) 0.5 a,b	7 (6,7) 0.5 a,b	7 (7,7) 0 a,b	6 (6,7) 0.5 a,b	0.022 B > C
Understand the potential time, type, and severity of complications to increase crisis awareness and improve motivation for self-care	7 (6,7) 0.5 a,b	7 (7,7) 0 a,b	7 (7,7) 0 a,b	6 (6,7) 0.5 a,b	0.119
Understand the symptoms and care approaches for acute and chronic complications	7 (6,7) 0.5 a,b	7 (7,7) 0 a,b	7 (7,7) 0 a,b	7 (6,7) 0.5 a,b	0.423
Understand the purpose of treatment or medication adjustment to increase compliance	7 (6,7) 0.5 a,b	7 (6,7) 0.5 a,b	7 (7,7) 0 a,b	7 (6,7) 0.5 a,b	0.799
Understand the effects of hormonal changes during puberty on glycemic control to reduce frustration	7 (6.5,7) 0.25 a,b	7 (7,7) 0 a,b	7 (7,7) 0 a,b	7 (6,7) 0.5 a,b	0.218
Understand the possible impact of pregnancy on glycemic control and clarify misconceptions	7 (6,7) 0.5 a,b	7 (6,7) 0.5 a,b	7 (7,7) 0 a,b	7 (6,7) 0.5 a,b	0.336
Understand contraceptive measures to reduce the impact of unintended conception in young girls in the context of maternal and child health	7 (6,7) 0.5 a,b	7 (6,7) 0.5 a,b	7 (7,7) 0 a,b	6 (6,7) 0.5 a,b	0.029 B > C
Establish accurate knowledge of disease inheritance and clarify misconceptions to avoid unnecessary stress and fear	7 (6,7) 0.5 a,b	7 (7,7) 0 a,b	7 (7,7) 0 a,b	6 (6,7) 0.5 a,b	0.012 A,B > C
Establish links to patient medical records that can be sent to other healthcare providers for reference when necessary or in case of an emergency	7 (6,7) 0.5 a,b	7 (6,7) 0.5 a,b	7 (7,7) 0 a,b	7 (6,7) 0.5 a,b	0.136
Discuss strategies to resist food cravings to improve the effectiveness of self-control with food	7 (6,7) 0.5 a,b	6 (5,7) 1 c,d	7 (7,7) 0 a,b	6 (6,7) 0.5 a,b	0.006 B > A,C
Understand the possible effects and impact of substance use on disease and health	7 (6,7) 0.5 a,b	7 (5,7) 1 c,d	7 (7,7) 0 a,b	6 (6,7) 0.5 a,b	0.076
**5. Healthcare dimension**					
Healthcare providers to replace accusations with gentle reminders and to avoid words that convey indifference and impatience	7 (6.5,7) 0.25 a,b	7 (6,7) 0.5 a,b	7 (7,7) 0 a,b	7 (6,7) 0.5 a,b	0.123
Understand the respective concerns and needs of the primary caregiver and the patient during physician consultation	7 (6,7) 0.5 a,b	7 (6,7) 0.5 a,b	7 (6,7) 0.5 a,b	7 (6,7) 0.5 a,b	0.799
Provide practice opportunities when delivering healthcare instructions	7 (6.5,7) 0.25 a,b	7 (6,7) 0.5 a,b	7 (7,7) 0 a,b	7 (6,7) 0.5 a,b	0.116
Provide private consultation space to discuss private issues	7 (6.5,7) 0.25 a,b	7 (7,7) 0 a,b	7 (6,7) 0.5 a,b	7 (6,7) 0.5 a,b	0.870
Provide healthcare guidance that meets the cognitive development and disease needs of patients of all ages	7(7,7) 0 a,b	7 (7,7) 0 a,b	7 (7,7) 0 a,b	7 (6,7) 0.5 a,b	0.265
Discuss topics on the transition from pediatric to adult care	7 (6,7) 0.5 a,b	7 (6,7) 0.5 a,b	7 (6,7) 0.5 a,b	7 (6,7) 0.5 a,b	0.804
Establish a multidisciplinary diagnosis and management plan and system to reduce the back and forth across departments	7 (7,7) 0 a,b	7 (7,7) 0 a,b	7 (7,7) 0 a,b	7 (6,7) 0.5 a,b	0.329
Provide and discuss domestic and foreign medical resources on type 1 diabetes	7 (6,7) 0.5 a,b	7 (6,7) 0.5 a,b	7 (6,7) 0.5 a,b	6 (5,7) 1 c,d	0.080
Provide free or subsidized regular full body examinations	7 (6,7) 0.5 a,b	7 (6,7) 0.5 a,b	7 (7,7) 0 a,b	6 (5,6) 0.5 b,c	0.006 B > C
Develop virtual healthcare to reduce the impact of medical treatment on work or study	7 (6,7) 0.5 a,b	7 (7,7) 0 a,b	7 (7,7) 0 a,b	6 (5,6) 0.5 b,c	0.001 A,B > C

a Level of importance: Q1 > 5, Q3 = 7 indicates “very important.” b Level of agreement: QD ≤ 0.6 indicates “high agreement.” c Level of importance: Q1 = 5, Q3 ≥ 6 indicates “important.” d Level of agreement: 0.6 < QD ≤ 1 indicates “moderate agreement”. A Group A: patients. B Group B: caregivers. C Group C: healthcare providers.

**Table 3:** Analysis of differences across stakeholder groups regarding healthcare needs for which a consensus on importance is not reached.

	TOTAL (*n* = 45)	GROUP A: Patients (*n* = 15)	GROUP B: Caregivers (*n* = 15)	GROUP C: Healthcare Providers (*n* = 15)	*p*-Value Post Hoc
	Median (Q1, Q3) QD	Median (Q1, Q3) QD	Median (Q1, Q3) QD	Median (Q1, Q3) QD	
**1. Technology dimension**					
Develop age-appropriate disease management apps or websites, such as interactive game-based designs	6 (5.5,7) 0.75a,d	6 (4,7) 1.5	7 (5,7) 1 c,d	6 (6,7) 0.5 a,b	0.695
Establish an anonymous patient community platform	7 (6,7) 0.5 a,b	6 (4,7) 1.5	7 (6,7) 0.5 a,b	6 (6,7) 0.5 a,b	0.176
Create a parent-only online chat room	6 (5,7) 1 c,d	5 (3,6) 1.5	7 (6,7) 0.5 a,b	6 (5,6) 0.5 b,c	0.009B > A
Create a chat room in which the patients can choose whether to share the contents with parents	6 (5,7) 1 c,d	6 (4,7) 1.5	7 (6,7) 0.5 a,b	5 (5,6) 0.5 b,c	0.013 B > C
**2. External support dimension**					
Provide more sharing opportunities among patients	7 (6,7) 0.5 a,b	7 (4,7) 1.5	7 (7,7) 0 a,b	7 (6,7) 0.5 a,b	0.073
Help develop interpersonal networks based on individual needs	6 (5,7) 1 c,d	6 (4,7) 1.5	7 (6,7) 0.5 a,b	6 (5,7) 1 c,d	0.065
Promote activities organized by diabetes associations through multiple channels	6 (5.5,7) 0.75 a,d	6 (4,7) 1.5	6 (6,7) 0.5 a,b	6 (6,7) 0.5 a,b	0.583
Provide appropriate subsidies	7 (6,7) 0.5 a,b	7 (6,7) 0.5 a,b	7 (7,7) 0 a,b	6 (6,6) 0b	0.003 B > C
Set up private friendly spaces in the public	7 (6,7) 0.5 a,b	7 (4,7) 1.5	7 (6,7) 0.5 a,b	7 (6,7) 0.5a,b	0.790
Create type 1 diabetes card for patients	6 (5,7) 1 c,d	5 (4,6) 1d	7 (6,7) 0.5 a,b	5 (5,6) 0.5 b,c	0.001 B > A,C
Change the name of the catastrophic illness card to something more positive to reduce labeling/stigmatization	6 (5,7) 1 c,d	6 (3,7) 2	7 (6,7) 0.5 a,b	6 (5,6) 0.5 b,c	0.024B > C
**3. Internal support dimension**					
Provide religious counseling resources	5 (4,6) 1d	4 (1,6) 2.5	6 (5,7) 1 c,d	6 (5,6) 0.5 b,c	0.040 C > A
Assess emotional distress and provide coping skills	6 (6,7) 0.5 a,b	6 (4,7) 1.5	7 (7,7) 0 a,b	6 (6,7) 0.5 a,b	0.012 B > A
Being understood for the fear and worry about death	6 (6,7) 0.5 a,b	6 (5,7) 1 c,b	7 (6,7) 0.5 a,b	6 (6,6) 0b	0.132
Organize spiritual support groups	6 (5,6.5) 0.75 c,d	5 (3,7) 2	6 (5,7) 1 c,d	6 (6,6) 0b	0.119

a Level of importance: Q1 > 5, Q3 = 7 indicates “very important.” b Level of agreement: QD ≤ 0.6 indicates “high agreement.” c Level of importance: Q1 = 5, Q3 ≥ 6 indicates “important.” d Level of agreement: 0.6 < QD ≤ 1 indicates “moderate agreement.” A Group A: patients. B Group B: caregivers. C Group C: healthcare providers.

## Data Availability

The data presented in this study are available on request from the corresponding author. The data are not publicly available due to confidentiality.

## Supplementary Materials

The following are available online at https://www.mdpi.com/article/10.3390/ijerph18137149/s1, Figure S1: The questionnaire of health care needs of patients with type 1 diabetes during the transition from adolescence to adulthood.
