# Supplementary material for: A Delphi Study on the Healthcare Needs of Patients with Type 1 Diabetes during the Transition from Adolescence to Adulthood: Consensus among Patients, Primary Caregivers, and Healthcare Providers
Source: Int J Environ Res Public Health. 2021 Jul 4;18(13):7149. doi: 10.3390/ijerph18137149 (PMC8296953; doi:10.3390/ijerph18137149)
Supplement: Supplementary file 1 [file ijerph-18-07149-s001.zip › ijerph-1239578-supplementary.pdf]

International Journal of
Environmental Research and Public Health

MDPI

Figure S1. **The questionnaire of health care needs of patients with type 1 diabetes during the transition from adolescence to adulthood**

Important: 1-not at all important, 2-low important, 3-slightly important, 4-neutral, 5-moderatoly important, 6-very important and 7-extremely important.

contents	important *							suggestions for revision
	1	2	3	4	5	6	7	
**1、Technology dimension**								
Develop a tailor-made app for type 1 diabetes	1	2	3	4	5	6	7	
Develop an app that can analyze the relationships between blood glucose changes, diet, and insulin dosage and provide recommendations	1	2	3	4	5	6	7	
Develop age-appropriate disease management apps or websites, such as interactive game-based designs	1	2	3	4	5	6	7	
Develop electronic diet and exercise journals	1	2	3	4	5	6	7	
Develop an electronic journal for instant recording of symptoms, scenarios, and management of acute complications	1	2	3	4	5	6	7	
Develop electronic guidelines on food calories and substitutions	1	2	3	4	5	6	7	
Establish an anonymous patient community platform	1	2	3	4	5	6	7	
Create a parent-only online chat room	1	2	3	4	5	6	7	
Create an anonymous chat room for physicians and patients	1	2	3	4	5	6	7	
Create a chat room in which the patients can choose whether to share the contents with parents	1	2	3	4	5	6	7	

Create an online portal for posting questions about type 1 diabetes	1	2	3	4	5	6	7	
Create a type 1 diabetes knowledge network	1	2	3	4	5	6	7	
other suggestions:								

**contents**	**important ***							**suggestions for revision**
	**1**	**2**	**3**	**4**	**5**	**6**	**7**	
**2、External support dimension**								
Provide organized and relevant information on type 1 diabetes	1	2	3	4	5	6	7	
Promote education on type 1 diabetes to reduce stigmatization of patients with type 1 diabetes due to public misunderstanding	1	2	3	4	5	6	7	
Need for a middleman to remind parents to learn to let go	1	2	3	4	5	6	7	
Hold seminars for parents to share the skills of letting go	1	2	3	4	5	6	7	
Mediate parent–child conflicts and enhance mutual understanding	1	2	3	4	5	6	7	
Provide patients with skills to communicate with parents	1	2	3	4	5	6	7	
Provide more sharing opportunities among patients	1	2	3	4	5	6	7	
Help develop interpersonal networks based on individual needs	1	2	3	4	5	6	7	
Provide employment counseling and consultation	1	2	3	4	5	6	7	

Hold employment seminars to share precautions and adjustment experience during job hunting and employment	1	2	3	4	5	6	7	
Promote activities organized by diabetes associations through multiple channels	1	2	3	4	5	6	7	
Diversify the activities organized by diabetes associations to meet the needs of patients from different age groups	1	2	3	4	5	6	7	
Provide appropriate subsidies	1	2	3	4	5	6	7	
Cover insulin pump supplies in the health insurance plan	1	2	3	4	5	6	7	
Set up private friendly spaces in the public	1	2	3	4	5	6	7	
Relax the disability handbook application criteria	1	2	3	4	5	6	7	
Include patients with type 1 diabetes under individuals with physical illness and provide learning assistance and resources	1	2	3	4	5	6	7	
Create type 1 diabetes card for patients	1	2	3	4	5	6	7	
Change the name of the catastrophic illness card to something more positive to reduce labeling/stigmatization	1	2	3	4	5	6	7	
other suggestions:								

**contents**	**important ***							**suggestions for revision**
	**1**	**2**	**3**	**4**	**5**	**6**	**7**	
**3、Internal support dimension**								
Provide religious counseling resources	1	2	3	4	5	6	7	
Assess emotional distress and provide coping skills	1	2	3	4	5	6	7	
Assess and treat stress-induced sleep disorders	1	2	3	4	5	6	7	
Being understood and accepted	1	2	3	4	5	6	7	
Being recognized and encouraged for personal improvement	1	2	3	4	5	6	7	
Being understood for the fear and worry about death	1	2	3	4	5	6	7	
Organize spiritual support groups	1	2	3	4	5	6	7	
Given appropriate autonomy to learn independence and responsibility	1	2	3	4	5	6	7	
Need for primary caregiver to replace control with supervision to reduce stress	1	2	3	4	5	6	7	
Provide resources for psychological counseling and consultation	1	2	3	4	5	6	7	
Provide stress-management strategies	1	2	3	4	5	6	7	
other suggestions:								

**contents**	**important ***							**suggestions for revision**
	**1**	**2**	**3**	**4**	**5**	**6**	**7**	
**4、Management dimension**								
Discuss the contents and goals of disease self-management	1	2	3	4	5	6	7	
Discuss how to integrate disease care into daily life	1	2	3	4	5	6	7	
Discuss diet-management strategies that meet developmental needs	1	2	3	4	5	6	7	
Discuss the accuracy of information on the internet	1	2	3	4	5	6	7	
Provide step-by-step disease self-care instructions based on individual conditions	1	2	3	4	5	6	7	
Plan and execute specific and feasible exercise programs based on patient preferences	1	2	3	4	5	6	7	
Enhance disease-related knowledge based on individual needs	1	2	3	4	5	6	7	
Differentiate between type 1 and type 2 diabetes and develop accurate understanding of own disease	1	2	3	4	5	6	7	
Understand changes in disease progression and increase awareness regarding health maintenance	1	2	3	4	5	6	7	
Understand the potential time, type, and severity of complications to increase crisis awareness and improve motivation for self-care	1	2	3	4	5	6	7	
Understand the symptoms and care approaches for acute and chronic complications	1	2	3	4	5	6	7	

Understand the purpose of treatment or medication adjustment to increase compliance	1	2	3	4	5	6	7	
Understand the effects of hormonal changes during puberty on glycemic control to reduce frustration	1	2	3	4	5	6	7	
Understand the possible impact of pregnancy on glycemic control and clarify misconception	1	2	3	4	5	6	7	
Understand contraceptive measures to reduce the impact of unintended conception in young girls in the context of maternal and child health	1	2	3	4	5	6	7	
Establish accurate knowledge of disease inheritance and clarify misconceptions to avoid unnecessary stress and fear	1	2	3	4	5	6	7	
Establish links to patient medical records that can be sent to other healthcare providers for reference when necessary or in case of an emergency								
Discuss strategies to resist food cravings to improve the effectiveness of self-control with food	1	2	3	4	5	6	7	
Understand the possible effects and impact of substance use on disease and health	1	2	3	4	5	6	7	
other suggestions:								

**contents**	**important ***							**suggestions for revision**
	**1**	**2**	**3**	**4**	**5**	**6**	**7**	
**5、 Healthcare dimension**								
1. Healthcare providers to replace accusations with gentle reminders and to avoid words that convey indifference and impatience	1	2	3	4	5	6	7	
2. Understand the respective concerns and needs of the primary caregiver and the patient during physician consultation	1	2	3	4	5	6	7	
3. Provide practice opportunities when delivering healthcare instructions†	1	2	3	4	5	6	7	
4. Provide private consultation space to discuss private issues	1	2	3	4	5	6	7	
5. Provide healthcare guidance that meets the cognitive development and disease needs of patients of all ages	1	2	3	4	5	6	7	
6. Discuss topics on the transition from pediatric to adult care	1	2	3	4	5	6	7	
7. Establish a multidisciplinary diagnosis and management plan and system to reduce the back and forth across departments	1	2	3	4	5	6	7	
8. Provide and discuss domestic and foreign medical resources on type 1 diabetes	1	2	3	4	5	6	7	
9. Provide free or subsidized regular full body examinations	1	2	3	4	5	6	7	
10. Develop virtual healthcare to reduce the impact of medical treatment on work or study	1	2	3	4	5	6	7	
other suggestions:								
